# Supplementary material for: Immune and sex-biased gene expression in the threatened Mojave desert tortoise, Gopherus agassizii
Source: PLoS One. 2020 Aug 26;15(8):e0238202. doi: 10.1371/journal.pone.0238202 (PMC7449761; doi:10.1371/journal.pone.0238202)
Supplement: S1 Table — *Denotes samples removed from analysis due to low sequencing depth. (DOCX) [file pone.0238202.s001.docx]

**Table S1 Sequencing and mapping statistics for the 25 samples included in this study.**

|  | Sequenced reads | Trimmed reads | % Reads retained | Unique mapped reads | % Unique mapped reads |
| --- | --- | --- | --- | --- | --- |
| CS0004* | 12,605,806 | 5,327,430 | 42 | 3,602,364 | 68 |
| CS0005 | 45,371,520 | 36,475,866 | 80 | 32,345,604 | 89 |
| CS0011* | 3,469,704 | 1,446,754 | 42 | 500,808 | 35 |
| CS0023 | 31,380,342 | 23,945,376 | 76 | 20,956,634 | 88 |
| CS0049 | 31,788,162 | 25,596,152 | 81 | 23,150,642 | 90 |
| CS0052 | 34,309,778 | 27,383,682 | 80 | 23,970,880 | 88 |
| CS0072 | 43,050,858 | 33,470,082 | 78 | 29,136,988 | 87 |
| CS0078 | 29,023,194 | 22,422,678 | 77 | 19,671,228 | 88 |
| CS0083 | 25,056,676 | 11,458,902 | 46 | 8,047,530 | 70 |
| 15780 | 31,248,310 | 24,898,158 | 80 | 21,910,144 | 88 |
| 21804* | 17,183,868 | 7,069,626 | 41 | 3,027,972 | 43 |
| 22003 | 24,723,088 | 17,590,034 | 71 | 14,412,992 | 82 |
| 22314* | 18,108,372 | 9,072,942 | 50 | 6,554,166 | 72 |
| 22335 | 30,636,910 | 24,545,682 | 80 | 21,538,414 | 88 |
| 22390 | 27,379,492 | 18,964,236 | 69 | 14,975,120 | 79 |
| 22399 | 25,532,200 | 21,203,860 | 83 | 18,953,728 | 89 |
| 18518 | 30,502,012 | 30,265,896 | 99 | 25,920,810 | 86 |
| 18602 | 33,746,568 | 27,823,844 | 82 | 24,988,610 | 90 |
| 18619 | 52,917,334 | 43,369,938 | 82 | 38,518,866 | 89 |
| 18789 | 46,148,660 | 37,626,428 | 82 | 32,864,176 | 87 |
| 19156 | 39,424,114 | 32,370,454 | 82 | 28,778,812 | 89 |
| 19431 | 24,725,394 | 15,903,008 | 64 | 13,049,796 | 82 |
| 19392 | 33,842,700 | 27,208,896 | 80 | 24,171,506 | 89 |
| 19730 | 31,055,390 | 24,411,460 | 79 | 21,849,496 | 90 |
| 21042 | 30,648,574 | 24,948,922 | 81 | 21,684,998 | 87 |

*Denotes samples removed from analysis due to low sequencing depth
