# Supplementary material for: Immune and sex-biased gene expression in the threatened Mojave desert tortoise, Gopherus agassizii
Source: PLoS One. 2020 Aug 26;15(8):e0238202. doi: 10.1371/journal.pone.0238202 (PMC7449761; doi:10.1371/journal.pone.0238202)
Supplement: S2 Table — (DOCX) [file pone.0238202.s002.docx]

**Table S2 Enriched Gene Ontology (GO) terms for Biological Processes that are uniquely differentially expressed based on puncture site.**

| **Adj. p value** | **GO ID** | **GO Term** | **No. of genes** | **Associated differentially expressed genes** |
| --- | --- | --- | --- | --- |
| 2.11E-02 | GO:0034112 | *positive regulation of homotypic cell-cell adhesion* | 1 | CCL5 |
| 2.11E-02 | GO:0071798 | *response to prostaglandin D* | 1 | PTGFR |
| 2.11E-02 | GO:0010628 | *positive regulation of gene expression* | 5 | HIF3A, PTGFR, ATF7IP, CCL5, UPF3A |
| 4.99E-02 | GO:0045766 | *positive regulation of angiogenesis* | 1 | CCL5 |
| 2.11E-02 | GO:0031622 | *positive regulation of fever generation* | 1 | CCL5 |
| 3.40E-02 | GO:0032270 | *positive regulation of cellular protein metabolic process* | 2 | CCL5, UPF3A |
| 1.71E-02 | GO:0009617 | *response to bacterium* | 4 | EPPIN, PTGFR, CLEC4E, CCL5 |
| 3.60E-02 | GO:0042110 | *T cell activation* | 2 | CLEC4E, CCL5 |
| 2.11E-02 | GO:0072677 | *eosinophil migration* | 1 | CCL5 |
| 2.11E-02 | GO:0006154 | *adenosine catabolic process* | 1 | ADAL |
| 3.89E-03 | GO:0051262 | *protein tetramerization* | 3 | CNGA2, TGM3, CCL5 |
| 3.40E-02 | GO:0042531 | *positive regulation of tyrosine phosphorylation of STAT protein* | 1 | CCL5 |
| 2.11E-02 | GO:0031269 | *pseudopodium assembly* | 1 | CCL5 |
| 1.71E-02 | GO:0035687 | *T-helper 1 cell extravasation* | 1 | CCL5 |
| 4.10E-02 | GO:0048661 | *positive regulation of smooth muscle cell proliferation* | 1 | CCL5 |
| 4.05E-02 | GO:0006734 | *NADH metabolic process* | 1 | MDH1B |
| 2.45E-02 | GO:0038094 | *Fc-gamma receptor signaling pathway* | 1 | CLEC4E |
| 2.45E-02 | GO:0046102 | *inosine metabolic process* | 1 | ADAL |
| 2.26E-02 | GO:0090026 | *positive regulation of monocyte chemotaxis* | 1 | CCL5 |
| 4.50E-02 | GO:0051899 | *membrane depolarization* | 1 | CNGA2 |
| 3.52E-02 | GO:0050708 | *regulation of protein secretion* | 2 | CLEC4E, CCL5 |
| 2.80E-02 | GO:0006108 | *malate metabolic process* | 1 | MDH1B |
| 2.11E-02 | GO:0043922 | *negative regulation by host of viral transcription* | 1 | CCL5 |
| 4.01E-02 | GO:0048522 | *positive regulation of cellular process* | 7 | HIF3A, PTGFR, CLEC4E, ATF7IP, PLPPR5, CCL5, UPF3A |
| 2.45E-02 | GO:0046103 | *inosine biosynthetic process* | 1 | ADAL |
| 2.56E-02 | GO:0042119 | *neutrophil activation* | 1 | CCL5 |
| 3.45E-02 | GO:0070098 | *chemokine-mediated signaling pathway* | 1 | CCL5 |
| 3.75E-02 | GO:0048739 | *cardiac muscle fiber development* | 1 | MYH11 |
| 2.06E-02 | GO:0045229 | *external encapsulating structure organization* | 1 | TGM3 |
| 3.57E-02 | GO:0048251 | *elastic fiber assembly* | 1 | MYH11 |
| 4.28E-02 | GO:0051928 | *positive regulation of calcium ion transport* | 1 | CCL5 |
| 3.40E-02 | GO:0045727 | *positive regulation of translation* | 1 | UPF3A |
| 2.11E-02 | GO:0060754 | *positive regulation of mast cell chemotaxis* | 1 | CCL5 |
| 1.71E-02 | GO:0035685 | *helper T cell diapedesis* | 1 | CCL5 |
| 2.11E-02 | GO:2000110 | *negative regulation of macrophage apoptotic process* | 1 | CCL5 |
| 2.11E-02 | GO:0036292 | *DNA rewinding* | 1 | ZRANB3 |
| 3.89E-03 | GO:0031584 | *activation of phospholipase D activity* | 2 | GNA13, CCL5 |
| 2.11E-02 | GO:0070234 | *positive regulation of T cell apoptotic process* | 1 | CCL5 |
| 2.06E-02 | GO:0098542 | *defense response to other organism* | 3 | EPPIN, CLEC4E, CCL5 |
| 4.05E-02 | GO:0042102 | *positive regulation of T cell proliferation* | 1 | CCL5 |
| 3.75E-02 | GO:0006099 | *tricarboxylic acid cycle* | 1 | MDH1B |
| 2.75E-02 | GO:0045672 | *positive regulation of osteoclast differentiation* | 1 | CCL5 |
| 3.57E-02 | GO:0045898 | *regulation of RNA polymerase II transcriptional preinitiation complex assembly* | 1 | ATF7IP |
| 2.06E-02 | GO:0033634 | *positive regulation of cell-cell adhesion mediated by integrin* | 1 | CCL5 |
| 3.40E-02 | GO:0006107 | *oxaloacetate metabolic process* | 1 | MDH1B |
| 2.11E-02 | GO:2000109 | *regulation of macrophage apoptotic process* | 1 | CCL5 |
| 3.83E-02 | GO:0071347 | *cellular response to interleukin-1* | 1 | CCL5 |
| 4.70E-02 | GO:0018149 | *peptide cross-linking* | 1 | TGM3 |
| 3.29E-02 | GO:0045071 | *negative regulation of viral genome replication* | 1 | CCL5 |
| 4.70E-02 | GO:0071356 | *cellular response to tumor necrosis factor* | 1 | CCL5 |
| 2.45E-02 | GO:1905523 | *positive regulation of macrophage migration* | 1 | CCL5 |
| 4.25E-02 | GO:0035315 | *hair cell differentiation* | 1 | TGM3 |
| 2.11E-02 | GO:2000341 | *regulation of chemokine (C-X-C motif) ligand 2 production* | 1 | CCL5 |
| 3.40E-02 | GO:0048478 | *replication fork protection* | 1 | ZRANB3 |
| 2.11E-02 | GO:0031652 | *positive regulation of heat generation* | 1 | CCL5 |
| 2.11E-02 | GO:0010820 | *positive regulation of T cell chemotaxis* | 1 | CCL5 |
| 3.40E-02 | GO:0014068 | *positive regulation of phosphatidylinositol 3-kinase signaling* | 1 | CCL5 |
| 2.11E-02 | GO:0071799 | *cellular response to prostaglandin D stimulus* | 1 | PTGFR |
| 3.40E-02 | GO:0045744 | *negative regulation of G-protein coupled receptor protein signaling pathway* | 1 | CCL5 |
| 3.20E-02 | GO:0006986 | *response to unfolded protein* | 1 | UPF3A |
| 3.60E-02 | GO:0001574 | *ganglioside biosynthetic process* | 1 | ST8SIA6 |
| 2.11E-02 | GO:0060753 | *regulation of mast cell chemotaxis* | 1 | CCL5 |
| 3.40E-02 | GO:0014911 | *positive regulation of smooth muscle cell migration* | 1 | CCL5 |
| 3.60E-02 | GO:0035584 | *calcium-mediated signaling using intracellular calcium source* | 1 | PTGFR |
| 2.11E-02 | GO:0001660 | *fever generation* | 1 | CCL5 |
