# Supplementary material for: Immune and sex-biased gene expression in the threatened Mojave desert tortoise, Gopherus agassizii
Source: PLoS One. 2020 Aug 26;15(8):e0238202. doi: 10.1371/journal.pone.0238202 (PMC7449761; doi:10.1371/journal.pone.0238202)
Supplement: S3 Table — (DOCX) [file pone.0238202.s003.docx]

**Table S3 Enriched Gene Ontology (GO) terms for Biological Processes that are uniquely differentially expressed among experimental groups (No Infection, NI; Medium Infection, MI; Severe Infection, SI).**

| **Adj. p value** | **GO ID** | **GO Term** | **No. of genes** | **Associated differentially expressed genes** |
| --- | --- | --- | --- | --- |
| 4.12E-02 | GO:1905168 | *positive regulation of double-strand break repair via homologous recombination* | 1 | MRNIP |
| 4.12E-02 | GO:0043101 | *purine-containing compound salvage* | 1 | HPRT1 |
| 4.12E-02 | GO:0046037 | *GMP metabolic process* | 1 | HPRT1 |
| 4.12E-02 | GO:0032764 | *negative regulation of mast cell cytokine production* | 1 | HMOX1 |
| 4.12E-02 | GO:0046084 | *adenine biosynthetic process* | 1 | HPRT1 |
| 4.12E-02 | GO:0039532 | *negative regulation of viral-induced cytoplasmic pattern recognition receptor signaling pathway* | 1 | RNF125 |
| 4.12E-02 | GO:0006168 | *adenine salvage* | 1 | HPRT1 |
| 4.12E-02 | GO:0034395 | *regulation of transcription from RNA polymerase II promoter in response to iron* | 1 | HMOX1 |
| 4.12E-02 | GO:0006807 | *nitrogen compound metabolic process* | 11 | SRI, SERINC1, MPG, MRNIP, HPRT1, KARS, LMO2, CDO1, RNF125, COPS8, BTG1 |
| 4.12E-02 | GO:0046101 | *hypoxanthine biosynthetic process* | 1 | HPRT1 |
| 4.12E-02 | GO:0019452 | *L-cysteine catabolic process to taurine* | 1 | CDO1 |
| 4.12E-02 | GO:0051279 | *regulation of release of sequestered calcium ion into cytosol* | 2 | SRI, CD19 |
| 4.12E-02 | GO:0039536 | *negative regulation of RIG-I signaling pathway* | 1 | RNF125 |
| 4.12E-02 | GO:0046038 | *GMP catabolic process* | 1 | HPRT1 |
| 4.12E-02 | GO:0009093 | *cysteine catabolic process* | 1 | CDO1 |
| 4.12E-02 | GO:0031325 | *positive regulation of cellular metabolic process* | 3 | LMO2, RNF125, COPS8 |
| 4.12E-02 | GO:0010524 | *positive regulation of calcium ion transport into cytosol* | 2 | SRI, CD19 |
| 4.12E-02 | GO:0009299 | *mRNA transcription* | 1 | LMO2 |
| 4.12E-02 | GO:0032480 | *negative regulation of type I interferon production* | 1 | RNF125 |
| 4.12E-02 | GO:0015959 | *diadenosine polyphosphate metabolic process* | 1 | KARS |
| 4.12E-02 | GO:1904220 | *regulation of serine C-palmitoyltransferase activity* | 1 | SERINC1 |
| 4.12E-02 | GO:0035162 | *embryonic hemopoiesis* | 1 | LMO2 |
| 4.12E-02 | GO:0000338 | *protein deneddylation* | 1 | COPS8 |
| 4.12E-02 | GO:0008285 | *negative regulation of cell proliferation* | 3 | KARS, COPS8, BTG1 |
| 4.12E-02 | GO:2000271 | *positive regulation of fibroblast apoptotic process* | 1 | BTG1 |
| 4.12E-02 | GO:0006430 | *lysyl-tRNA aminoacylation* | 1 | KARS |
| 4.12E-02 | GO:0006178 | *guanine salvage* | 1 | HPRT1 |
| 4.12E-02 | GO:0043392 | *negative regulation of DNA binding* | 2 | SRI, HMOX1 |
| 4.12E-02 | GO:0010033 | *response to organic substance* | 6 | SRI, HPRT1, KARS, LMO2, CDO1, RNF125 |
| 4.12E-02 | GO:1904427 | *positive regulation of calcium ion transmembrane transport* | 2 | SRI, CD19 |
| 4.12E-02 | GO:0002276 | *basophil activation involved in immune response* | 1 | KARS |
| 4.12E-02 | GO:0033762 | *response to glucagon* | 1 | CDO1 |
| 4.12E-02 | GO:1901844 | *regulation of cell communication by electrical coupling involved in cardiac conduction* | 1 | SRI |
| 4.12E-02 | GO:0006166 | *purine ribonucleoside salvage* | 1 | HPRT1 |
| 4.12E-02 | GO:0097066 | *response to thyroid hormone* | 1 | LMO2 |
| 4.12E-02 | GO:0051281 | *positive regulation of release of sequestered calcium ion into cytosol* | 2 | SRI, CD19 |
| 4.12E-02 | GO:1901224 | *positive regulation of NIK/NF-kappaB signaling* | 1 | COPS8 |
| 4.12E-02 | GO:0039529 | *RIG-I signaling pathway* | 1 | RNF125 |
| 4.12E-02 | GO:0015960 | *diadenosine polyphosphate biosynthetic process* | 1 | KARS |
| 4.12E-02 | GO:0045647 | *negative regulation of erythrocyte differentiation* | 1 | LMO2 |
| 4.12E-02 | GO:0006788 | *heme oxidation* | 1 | HMOX1 |
| 4.12E-02 | GO:0002275 | *myeloid cell activation involved in immune response* | 2 | HMOX1, KARS |
| 4.12E-02 | GO:0002718 | *regulation of cytokine production involved in immune response* | 2 | HMOX1, KARS |
| 4.24E-02 | GO:0031349 | *positive regulation of defense response* | 2 | KARS, RNF125 |
| 4.24E-02 | GO:0006658 | *phosphatidylserine metabolic process* | 1 | SERINC1 |
| 4.24E-02 | GO:0010212 | *response to ionizing radiation* | 2 | MRNIP, KARS |
| 4.24E-02 | GO:0072718 | *response to cisplatin* | 1 | HMOX1 |
| 4.24E-02 | GO:0009410 | *response to xenobiotic stimulus* | 2 | HPRT1, CDO1 |
| 4.24E-02 | GO:1905050 | *positive regulation of metallopeptidase activity* | 1 | KARS |
| 4.24E-02 | GO:0046040 | *IMP metabolic process* | 1 | HPRT1 |
| 4.27E-02 | GO:1901841 | *regulation of high voltage-gated calcium channel activity* | 1 | SRI |
| 4.65E-02 | GO:0002252 | *immune effector process* | 3 | HPRT1, KARS, RNF125 |
| 4.65E-02 | GO:0043305 | *negative regulation of mast cell degranulation* | 1 | HMOX1 |
| 4.65E-02 | GO:0055118 | *negative regulation of cardiac muscle contraction* | 1 | SRI |
| 4.65E-02 | GO:0045603 | *positive regulation of endothelial cell differentiation* | 1 | BTG1 |
| 4.72E-02 | GO:0060315 | *negative regulation of ryanodine-sensitive calcium-release channel activity* | 1 | SRI |
| 4.74E-02 | GO:0090160 | *Golgi to lysosome transport* | 1 | CCDC91 |
| 4.82E-02 | GO:0010759 | *positive regulation of macrophage chemotaxis* | 1 | KARS |
| 4.92E-02 | GO:0045663 | *positive regulation of myoblast differentiation* | 1 | BTG1 |
| 4.92E-02 | GO:0042994 | *cytoplasmic sequestering of transcription factor* | 1 | SRI |
| 4.98E-02 | GO:1900745 | *positive regulation of p38MAPK cascade* | 1 | KARS |
