# Supplementary material for: Immune and sex-biased gene expression in the threatened Mojave desert tortoise, Gopherus agassizii
Source: PLoS One. 2020 Aug 26;15(8):e0238202. doi: 10.1371/journal.pone.0238202 (PMC7449761; doi:10.1371/journal.pone.0238202)
Supplement: S4 Table — (DOCX) [file pone.0238202.s004.docx]

**Table S4 Enriched KEGG pathways for genes uniquely differentially expressed based on sex.**

| **Adj. p value** | **KEGG ID** | **KEGG Term** | **No. of genes** | **Associated differentially expressed genes** |
| --- | --- | --- | --- | --- |
| 3.61E-04 | KEGG:01524 | *Platinum drug resistance* | 7 | PDPK1, PIK3R3, FADD, BIRC3, ATP7A, MAPK1, CYCS |
| 1.38E-03 | KEGG:04114 | *Oocyte meiosis* | 10 | PPP1CB, PPP2R5C, YWHAB, YWHAH, CDK1, PPP3CB, SLK, MAD2L1, PPP3R1, MAPK1 |
| 1.38E-03 | KEGG:05203 | *Viral carcinogenesis* | 11 | GTF2H1, MAPKAPK2, YWHAB, CDK1, GTF2A1, USP7, UBE3A, PIK3R3, RBPJ, PIK3R1, MAPK1 |
| 1.38E-03 | KEGG:05169 | *Epstein-Barr virus infection* | 13 | POLR3D, YWHAB, YWHAH, HDAC2, CDK1, PSMC6, USP7, PSMD14, POLR3C, PIK3R3, RBPJ, PIK3R1, CSNK2A1 |
| 3.17E-03 | KEGG:04141 | *Protein processing in endoplasmic reticulum* | 18 | SEC24B, DNAJB1, DERL3, UBE2G2, NFE2L2, HSP90B1, HSP90AA1, SSR1, DNAJC3, PDIA4, HSPA5, SEC62, PLAA, EIF2AK1, EIF2AK3, DNAJA2, SEC24A, NPLOC4 |
| 3.17E-03 | KEGG:05215 | *Prostate cancer* | 7 | HSP90B1, HSP90AA1, PDPK1, ZEB1, PIK3R3, PIK3R1, MAPK1 |
| 3.17E-03 | KEGG:05164 | *Influenza A* | 15 | TRIM25, DNAJB1, KPNA2, XPO1, IFNAR1, IFIH1, PIK3R3, ACTB, EIF2AK3, OAS3, HNRNPUL1, PIK3R1, NXT2, MAPK1, CYCS |
| 4.18E-03 | KEGG:04216 | *Ferroptosis* | 3 | TFRC, NCOA4, PCBP2 |
| 5.41E-03 | KEGG:04144 | *Endocytosis* | 23 | AP2A2, RAB8A, KIF5B, GIT1, CYTH1, RAB5A, VPS26A, RUFY1, TFRC, USP8, CHMP5, SH3GL3, WAS, VPS35, ARAP1, SNX2, PRKCI, WIPF2, CLTC, ARF1, STAM2, ARFGEF2, GRK6 |
| 5.41E-03 | KEGG:04370 | *VEGF signaling pathway* | 7 | RAC1, MAPKAPK2, PPP3CB, PIK3R3, PPP3R1, PIK3R1, MAPK1 |
| 5.41E-03 | KEGG:04722 | *Neurotrophin signaling pathway* | 7 | MAPKAPK2, PSEN1, PDPK1, PIK3R3, PIK3R1, MAPK1, ARHGDIG |
| 5.41E-03 | KEGG:04140 | *Autophagy - animal* | 13 | PPP2CA, RPS6KB1, PDPK1, MLST8, RRAGD, PIK3R3, EIF2AK3, ATG2B, PIK3R1, ATG16L2, MAPK1, HMGB1, NRBF2 |
| 5.41E-03 | KEGG:03015 | *mRNA surveillance pathway* | 12 | RNMT, PABPC4, PPP1CB, PPP2R5C, PPP2CA, PPP2R5E, ACIN1, ETF1, NCBP1, UPF3B, PPP2R2D, NXT2 |
| 5.41E-03 | KEGG:04914 | *Progesterone-mediated oocyte maturation* | 4 | CDK1, HSP90AA1, PIK3R3, MAPK1 |
| 5.41E-03 | KEGG:05165 | *Human papillomavirus infection* | 29 | DVL3, IKBKG, HDAC5, PPP2R5C, HDAC2, PSEN1, PPP2CA, RPS6KB1, PPP2R5E, SCRIB, IFNAR1, HDAC3, TCF7L2, UBE3A, LAMC1, RBL1, PIK3R3, FADD, ATP6V1H, ITGA2B, PRKCI, ATP6V0A2, RBPJ, PIK3R1, PPP2R2D, ITGA1, PATJ, MAPK1, RPS6KB2 |
| 5.41E-03 | KEGG:04530 | *Tight junction* | 16 | MARVELD3, RAC1, RAB8A, ACTR2, PPP2CA, HSPA4, SCRIB, ACTR3, ACTB, WAS, MYL12B, RAPGEF6, PRKCI, PPP2R2D, PATJ, ERBB2 |
| 6.24E-03 | KEGG:04210 | *Apoptosis* | 6 | PDPK1, PIK3R3, FADD, BIRC3, MAPK1, CYCS |
| 7.74E-03 | KEGG:04218 | *Cellular senescence* | 7 | PPP1CB, MAPKAPK2, CDK1, PIK3R3, PPP3R1, LIN54, MAPK1 |
| 8.02E-03 | KEGG:04910 | *Insulin signaling pathway* | 10 | PPP1CB, RPS6KB1, PRKAR1A, PDPK1, PTPN1, EIF4E, PIK3R3, PKLR, PIK3R1, MAPK1 |
| 8.13E-03 | KEGG:04120 | *Ubiquitin mediated proteolysis* | 14 | CUL3, UBE2G2, BRCA1, HERC4, FBXW11, CDC34, HUWE1, UBE3A, CUL4A, BIRC3, CUL5, PIAS1, UBA6, UBA2 |
| 8.13E-03 | KEGG:05170 | *Human immunodeficiency virus 1 infection* | 8 | CDK1, RPS6KB1, PIK3R3, FADD, CUL4A, PPP3R1, MAPK1, CYCS |
| 8.13E-03 | KEGG:03050 | *Proteasome* | 4 | PSMC6, PSMD14, POMP, PSMA4 |
| 8.13E-03 | KEGG:05014 | *Amyotrophic lateral sclerosis (ALS)* | 6 | RAC1, RAB5A, PPP3CB, CAT, PPP3R1, CYCS |
| 8.13E-03 | KEGG:04960 | *Aldosterone-regulated sodium reabsorption* | 4 | PDPK1, PIK3R3, PIK3R1, MAPK1 |
| 8.13E-03 | KEGG:05212 | *Pancreatic cancer* | 10 | RAC1, IKBKG, RALA, RPS6KB1, PIK3R3, BRCA2, PIK3R1, ERBB2, MAPK1, RPS6KB2 |
| 8.13E-03 | KEGG:04110 | *Cell cycle* | 8 | DBF4, YWHAB, YWHAH, HDAC2, CDK1, CDKN2C, MAD2L1, TFDP2 |
| 8.13E-03 | KEGG:04520 | *Adherens junction* | 8 | RAC1, CSNK2B, PTPN1, ACTB, WAS, ERBB2, MAPK1, CSNK2A1 |
| 8.13E-03 | KEGG:05231 | *Choline metabolism in cancer* | 6 | RPS6KB1, PDPK1, PIK3R3, DGKZ, PIK3R1, MAPK1 |
| 1.12E-02 | KEGG:03008 | *Ribosome biogenesis in eukaryotes* | 9 | XPO1, CSNK2B, RBM28, MPHOSPH10, XRN1, TBL3, NXT2, CSNK2A1, RIOK2 |
| 1.16E-02 | KEGG:05161 | *Hepatitis B* | 5 | YWHAB, PIK3R3, FADD, MAPK1, CYCS |
| 1.16E-02 | KEGG:03040 | *Spliceosome* | 14 | PUF60, PRPF31, EFTUD2, DDX46, ACIN1, PLRG1, NCBP1, SRSF10, SYF2, SRSF9, USP39, PPIH, PRPF4, SF3B4 |
| 1.16E-02 | KEGG:04215 | *Apoptosis - multiple species* | 3 | FADD, BIRC3, CYCS |
| 1.16E-02 | KEGG:04931 | *Insulin resistance* | 6 | PPP1CB, RPS6KB1, PDPK1, PTPN1, PIK3R3, PIK3R1 |
| 1.16E-02 | KEGG:04213 | *Longevity regulating pathway - multiple species* | 5 | HDAC2, RPS6KB1, CAT, PIK3R3, PIK3R1 |
| 1.49E-02 | KEGG:05100 | *Bacterial invasion of epithelial cells* | 7 | RAC1, PIK3R3, ACTB, WAS, PIK3R1, CLTC, RHOG |
| 1.52E-02 | KEGG:05205 | *Proteoglycans in cancer* | 8 | TFAP4, PPP1CB, RPS6KB1, PDPK1, FLNB, PIK3R3, PIK3R1, MAPK1 |
| 1.56E-02 | KEGG:05210 | *Colorectal cancer* | 10 | RAC1, MSH6, RALA, RPS6KB1, TCF7L2, PIK3R3, PIK3R1, MAPK1, CYCS, RPS6KB2 |
| 1.64E-02 | KEGG:04657 | *IL-17 signaling pathway* | 5 | HSP90B1, HSP90AA1, FADD, MAPK6, MAPK1 |
| 1.68E-02 | KEGG:00520 | *Amino sugar and nucleotide sugar metabolism* | 4 | TSTA3, GNPDA2, AMDHD2, GNPNAT1 |
| 1.68E-02 | KEGG:05132 | *Salmonella infection* | 7 | RAC1, FLNB, ACTB, WAS, RILP, MAPK1, RHOG |
| 1.82E-02 | KEGG:04330 | *Notch signaling pathway* | 5 | DVL3, HDAC2, PSEN1, RBPJ, DTX3L |
| 1.95E-02 | KEGG:04510 | *Focal adhesion* | 12 | RAC1, PPP1CB, PDPK1, FLNB, PIK3R3, ACTB, BIRC3, ITGA2B, MYL12B, PIK3R1, ERBB2, MAPK1 |
| 1.95E-02 | KEGG:05213 | *Endometrial cancer* | 4 | PDPK1, PIK3R3, PIK3R1, MAPK1 |
| 1.95E-02 | KEGG:04066 | *HIF-1 signaling pathway* | 7 | RPS6KB1, TFRC, EIF4E, PIK3R3, PIK3R1, ERBB2, MAPK1 |
| 1.95E-02 | KEGG:05168 | *Herpes simplex infection* | 14 | PPP1CB, CDK1, HCFC2, CDC34, USP7, IFNAR1, CSNK2B, TAF5, IFIH1, FADD, EIF2AK3, OAS3, CYCS, CSNK2A1 |
| 2.03E-02 | KEGG:05160 | *Hepatitis C* | 12 | IKBKG, PPP2CA, IFNAR1, PDPK1, PIK3R3, EIF2AK1, EIF2AK3, PIAS1, OAS3, PIK3R1, PPP2R2D, MAPK1 |
| 2.03E-02 | KEGG:03410 | *Base excision repair* | 5 | POLE2, POLL, SMUG1, LIG1, HMGB1 |
| 2.03E-02 | KEGG:01521 | *EGFR tyrosine kinase inhibitor resistance* | 6 | RPS6KB1, EIF4E, PIK3R3, PIK3R1, ERBB2, MAPK1 |
| 2.03E-02 | KEGG:04390 | *Hippo signaling pathway* | 11 | DVL3, PPP1CB, YWHAB, YWHAH, FBXW11, PPP2CA, SCRIB, ACTB, PPP2R2D, MOB1A, PATJ |
| 2.10E-02 | KEGG:04068 | *FoxO signaling pathway* | 6 | USP7, PDPK1, CAT, PIK3R3, PIK3R1, MAPK1 |
| 2.11E-02 | KEGG:03013 | *RNA transport* | 14 | PHAX, PABPC4, NUP50, XPO1, ACIN1, RANGAP1, DDX20, EIF4E, NCBP1, UPF3B, NXT2, THOC7, EIF2S2, EIF4EBP3 |
| 2.11E-02 | KEGG:05167 | *Kaposi sarcoma-associated herpesvirus infection* | 6 | MAPKAPK2, PIK3R3, FADD, PPP3R1, MAPK1, CYCS |
| 2.11E-02 | KEGG:04662 | *B cell receptor signaling pathway* | 6 | RAC1, PPP3CB, PIK3R3, PPP3R1, PIK3R1, MAPK1 |
| 2.11E-02 | KEGG:04919 | *Thyroid hormone signaling pathway* | 6 | HDAC2, PDPK1, PIK3R3, MED13, PIK3R1, MAPK1 |
| 2.16E-02 | KEGG:04660 | *T cell receptor signaling pathway* | 5 | PDPK1, PIK3R3, PPP3R1, PIK3R1, MAPK1 |
| 2.33E-02 | KEGG:03460 | *Fanconi anemia pathway* | 7 | BRCA1, POLH, ATRIP, BLM, BRCA2, FANCB, PMS2 |
| 2.38E-02 | KEGG:04664 | *Fc epsilon RI signaling pathway* | 4 | PDPK1, PIK3R3, PIK3R1, MAPK1 |
| 2.45E-02 | KEGG:05223 | *Non-small cell lung cancer* | 3 | PDPK1, PIK3R3, MAPK1 |
| 2.57E-02 | KEGG:04625 | *C-type lectin receptor signaling pathway* | 7 | RELB, MAPKAPK2, PPP3CB, PIK3R3, PPP3R1, PIK3R1, MAPK1 |
| 2.58E-02 | KEGG:01522 | *Endocrine resistance* | 5 | RPS6KB1, CDKN2C, PIK3R3, PIK3R1, MAPK1 |
| 2.59E-02 | KEGG:03450 | *Non-homologous end-joining* | 3 | RAD50, POLL, MRE11 |
| 2.60E-02 | KEGG:05221 | *Acute myeloid leukemia* | 4 | RPS6KB1, PIK3R3, PIK3R1, MAPK1 |
| 2.60E-02 | KEGG:04151 | *PI3K-Akt signaling pathway* | 16 | RAC1, PPP2R5C, YWHAB, YWHAH, HSP90B1, PPP2CA, RPS6KB1, HSP90AA1, PDPK1, EIF4E, PIK3R3, ITGA2B, PIK3R1, PPP2R2D, ERBB2, MAPK1 |
| 2.60E-02 | KEGG:05010 | *Alzheimer disease* | 6 | PSEN1, UQCRC2, FADD, PPP3R1, MAPK1, CYCS |
| 2.63E-02 | KEGG:04071 | *Sphingolipid signaling pathway* | 8 | RAC1, PPP2R5C, PPP2CA, PDPK1, PIK3R3, PIK3R1, PPP2R2D, MAPK1 |
| 2.63E-02 | KEGG:04152 | *AMPK signaling pathway* | 11 | RAB8A, PPP2R5C, PPP2CA, RPS6KB1, PPP2R5E, HMGCR, PDPK1, PIK3R3, PIK3R1, PPP2R2D, RPS6KB2 |
| 2.81E-02 | KEGG:04668 | *TNF signaling pathway* | 5 | PIK3R3, FADD, BIRC3, PIK3R1, MAPK1 |
| 2.82E-02 | KEGG:04115 | *p53 signaling pathway* | 2 | CDK1, CYCS |
| 2.87E-02 | KEGG:05216 | *Thyroid cancer* | 2 | NCOA4, MAPK1 |
| 2.87E-02 | KEGG:05145 | *Toxoplasmosis* | 4 | PDPK1, BIRC3, MAPK1, CYCS |
| 3.00E-02 | KEGG:03022 | *Basal transcription factors* | 4 | GTF2F1, GTF2H1, GTF2A1, TAF5 |
| 3.08E-02 | KEGG:03420 | *Nucleotide excision repair* | 3 | GTF2H1, POLE2, CUL4A |
| 3.15E-02 | KEGG:04142 | *Lysosome* | 3 | LITAF, NPC1, ATP6V0A2 |
| 3.43E-02 | KEGG:05152 | *Tuberculosis* | 5 | FADD, PPP3R1, ATP6V0A2, MAPK1, CYCS |
| 3.43E-02 | KEGG:04915 | *Estrogen signaling pathway* | 4 | HSP90B1, HSP90AA1, PIK3R3, MAPK1 |
| 4.19E-02 | KEGG:05225 | *Hepatocellular carcinoma* | 14 | SMARCB1, DVL3, NFE2L2, RPS6KB1, SMARCD1, TCF7L2, SMARCC2, ACTL6A, PIK3R3, ACTB, PIK3R1, MAPK1, SMARCD2, RPS6KB2 |
| 4.36E-02 | KEGG:04012 | *ErbB signaling pathway* | 4 | RPS6KB1, PIK3R3, PIK3R1, MAPK1 |
| 4.37E-02 | KEGG:04930 | *Type II diabetes mellitus* | 2 | PIK3R3, MAPK1 |
| 4.42E-02 | KEGG:04666 | *Fc gamma R-mediated phagocytosis* | 4 | RPS6KB1, PIK3R3, PIK3R1, MAPK1 |
