# Supplementary figures and images for: Immune and sex-biased gene expression in the threatened Mojave desert tortoise, Gopherus agassizii
Source: PLoS One. 2020 Aug 26;15(8):e0238202. doi: 10.1371/journal.pone.0238202 (PMC7449761; doi:10.1371/journal.pone.0238202)

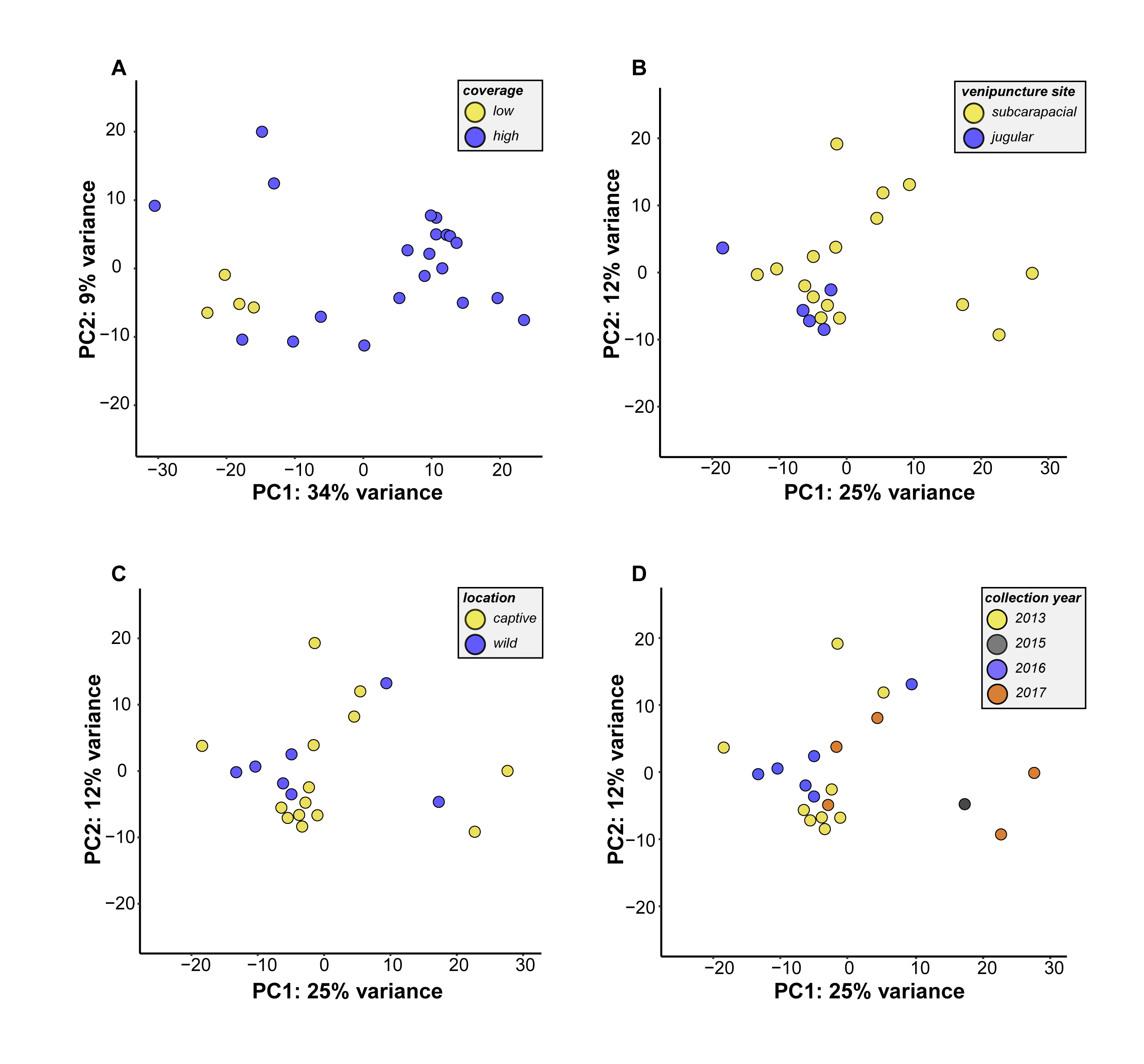

Supplement: S1 Fig — Color-coded by additional variables relevant to the experimental design: (A) low coverage samples, (B) venipuncture site, (C) wild vs. captive, (D) collection year. Only high coverage samples are shown for venipuncture site, wild vs. captive, and collection year. Venipuncture site showed a pattern and was analyzed through the DESeq2 pipeline (see S2 and S3 Figs, and S1 Appendix). (TIF) [file pone.0238202.s005.tif]

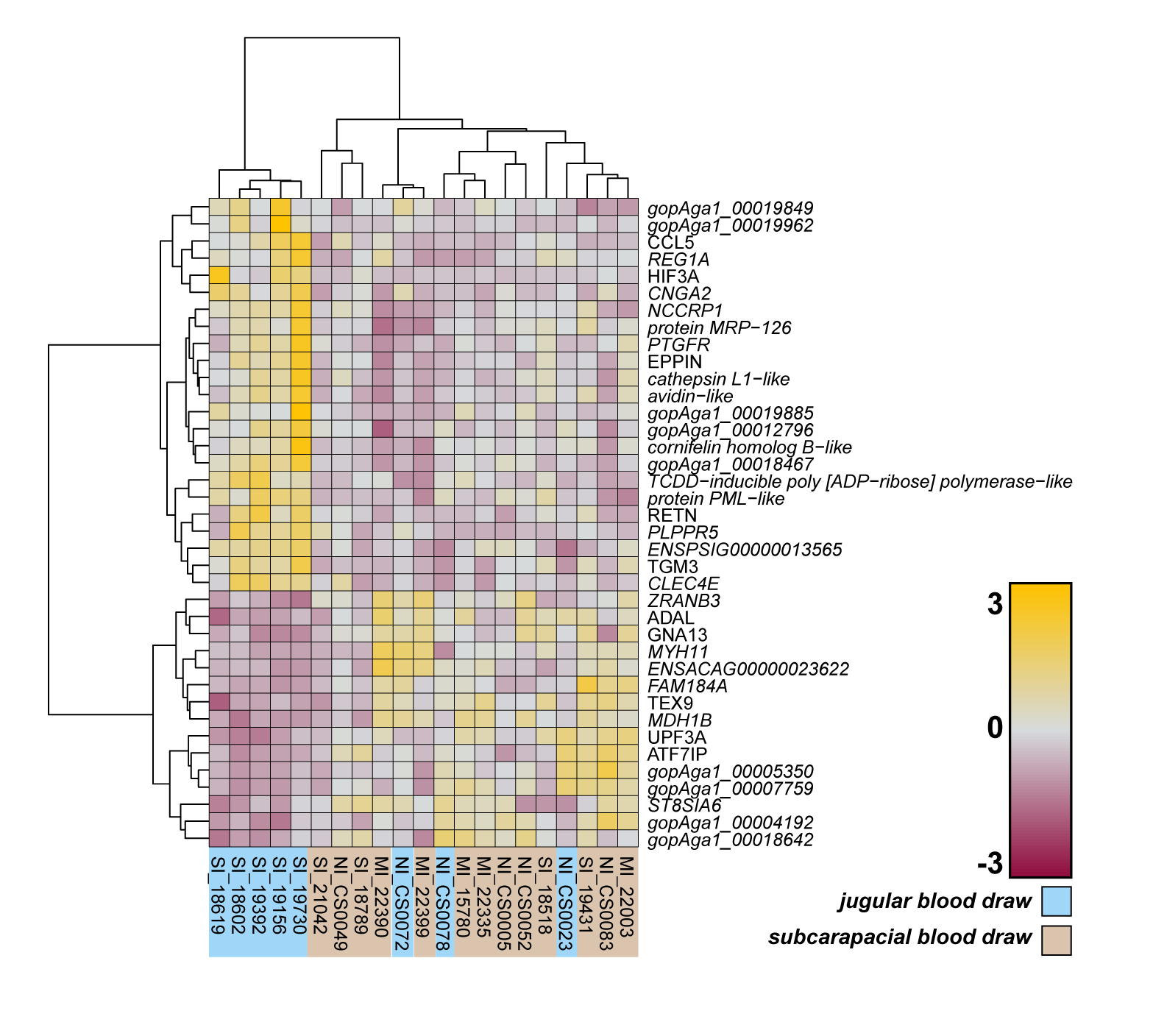

Supplement: S2 Fig — Subcarapacial blood draws may have aspirated lymph fluid due to proximal lymphatic vessels, however this is the preferred phlebotomy technique by management agencies. Expression values are mean-centered, regularized log counts and colors are represented as z-score values. Jugular, light blue; subcarapacial, tan. (TIF) [file pone.0238202.s006.tif]

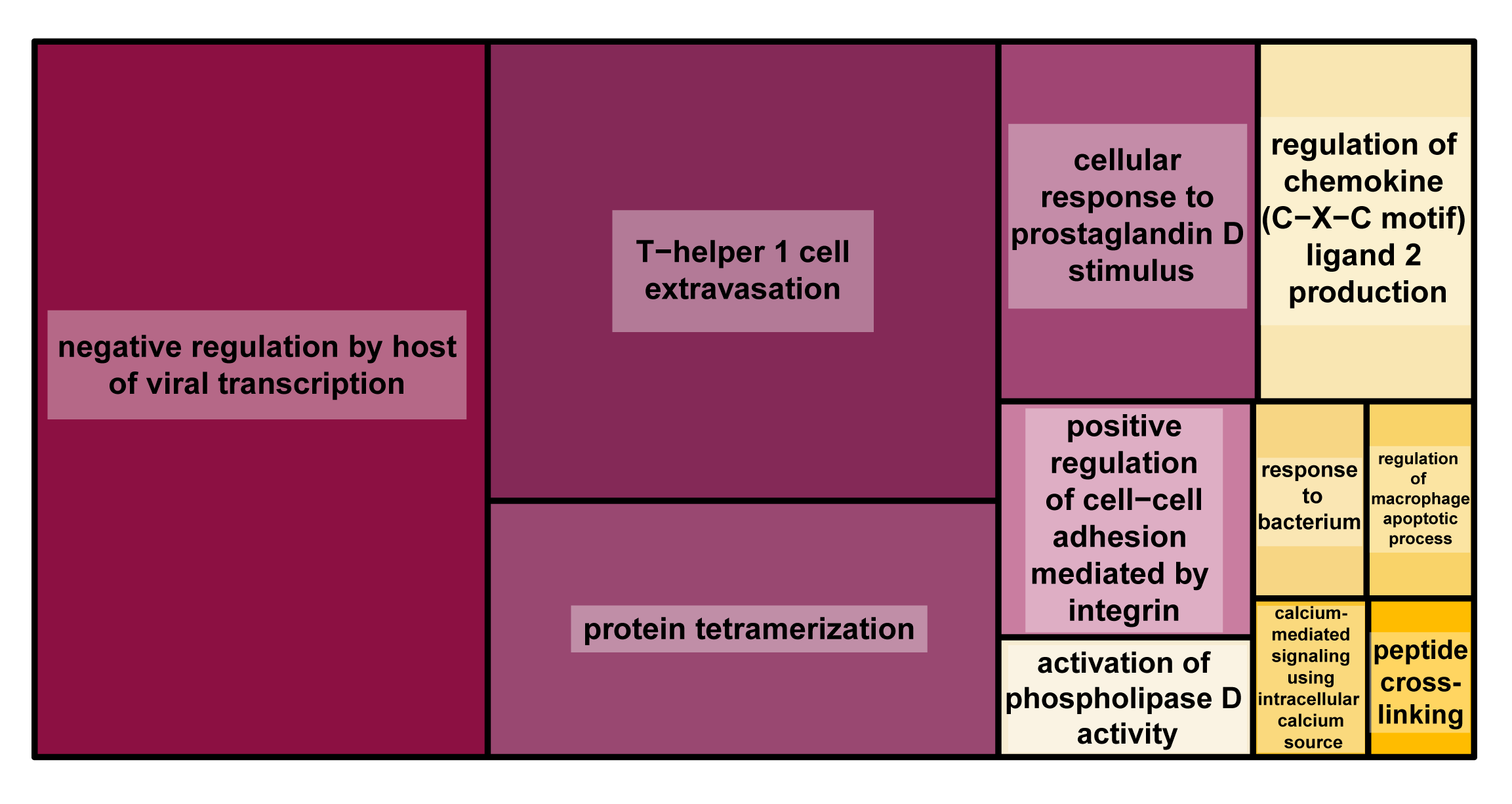

Supplement: S3 Fig — (TIF) [file pone.0238202.s007.tif]
