# Supplementary material for: Immune and sex-biased gene expression in the threatened Mojave desert tortoise, Gopherus agassizii
Source: PLoS One. 2020 Aug 26;15(8):e0238202. doi: 10.1371/journal.pone.0238202 (PMC7449761; doi:10.1371/journal.pone.0238202)
Supplement: Attachment — Submitted filename: Xu Dolby_Editor and Reviewer Comments_FINAL.docx [file pone.0238202.s012.docx]

**Response to Manuscript Reviewers**

**Manuscript**: Immune and sex-biased gene expression in the threatened Mojave desert tortoise, *Gopherus agassizii*.

**Authors:** Cindy Xu, Greer A. Dolby, K. Kristina Drake, Todd C. Esque, and Kenro Kusumi.

In Review with PLOS One.

**Response to Internal and Journal Reviewers:**

Thank you for your time and editorial suggestions to improve the clarity and content of this manuscript. We incorporated your suggestions and reworded sentence structure and supporting information as needed. We have addressed your specific comments and questions below.

Sincerely,

Cindy Xu, Greer A. Dolby, K. Kristina Drake, Todd C. Esque, and Kenro Kusumi ____________________________________________________________________________

**Comments Received from USGS Internal Reviewer** on May 6, 2020.

Elizabeth Hunter, PhD; Georgia Southern University

I finished the review of your paper.  I thought it was well designed (all potential design issues, such as confounding variables, were taken care of), addressed important questions for the basic biology and conservation of this species (and likely other reptiles), and presented very interesting results.  Most of all, I thought it was exceptionally well written.  I was anticipating having to struggle through it as a person not well-versed in these transcription types of studies, but I found it quite easy to understand, and I learned quite a lot!  Very nice paper! I have some minor questions/suggestions in the track changes of the attached word document.  Good luck with the journal reviews.

- [**Line 54**] “If sex differences are going to be a focus of the paper, it might be good to very briefly outline what is known in mammalian systems (to set up the expectation and see how it differs in non-avian reptiles)”. Response: We included a new paragraph on sex-biased gene expression in the introduction.
- [**Line 83**] “Were the severe infection tortoises also inoculated, or were they discovered with a severe infection?” Response: The infected tortoises were discovered infected and were not inoculated from experiments. We added the following to the Experimental Design section in methods, “1) individuals discovered with severe *M. agassizii* infection and were not inoculated from experimentation (Severe Infection group, SI);” on line 211-212 of the track-change version and on line 109-110 of the clean version.
- **[Line 107]** tortoises – “also adults”? Response: We edited this sentence for clarity.
- **[Line 117]** Can you give some indication about whether conditions experienced by the tortoises among these years might have been similar or different? I.e., was it a particularly dry or wet year in any of these years? Response: The control (NI) tortoises were wild tortoises from a site north of Las Vegas, the low-infection (LI) and severe-infection (SI) tortoises were captive animals housed in Las Vegas. Although the samples were collected in different years as indicated in the methods, we would only be speculating on the potential impact that precipitation may have, if any. During the month of sample for all groups, the precipitation was fairly similar. However, captive tortoises received regular food and water throughout their captivity. We addressed this in the methods to clarify. We hope to address the potential role of hydration status in future studies.
- **[Line 167]** Was there any confounding of puncture site with treatment? In other words, were only the wild tortoises sampled with subcarapacial (for example)? You might give an idea of the number of individuals sampled with each method in each treatment group. Response: We added this information to the methods for clarity. “All MI and NI tortoises were sampled using subcarapacial venipuncture while SI tortoises were sampled using both subcarapacial and jugular venipuncture (N=9, 4 subcarapacial:5 jugular).”
- **[Line 226]** Captivity vs. wild was definitely one I was wondering about. You might considering including this figure in the main text, as I think it’s important to rule out this potentially confounding variable. Response: We agree with the reviewer that captive vs. wild animals is an important variable to consider but did not find it to be a confounding variable in this study. We include the PCA analysis of animal location in Figure S1.
- **[Line 241]** Agreed. This is really important to know. Response: We thank the reviewer for her concurrence.
- **[Line 259]** Can you explain the color legend that ranges from -3 to 3? I really liked the graphical schematics – really helped with interpretation. Response: We revised the figure legend in Figure 2,4 and S2 to improve clarity. To the figure captions we added that the color represents the level of gene expression.
- **[Line 297]** Same comment on the color legend. It seems there are two main groups in this figure (indicated by the tree diagram). Do those groups have any meaning, or is it just some genes are more expressed in females and some are more expressed in males? Response. The tree diagram shows differentially expressed genes, which are clustered by similar expression patterns. The top cluster are genes upregulated in females and downregulated in males while the bottom cluster are genes downregulated in females but upregulated in males. To the figure captions we added that the color represents the level of gene expression.
- **[Line 390]** This is fascinating all on its own. Response: Thank you.
- **[Line 400]** Is there any indication in the literature that males have higher mortality rates or more severe symptoms from M. agassizii? It would be interesting to connect this immune response to those outcomes. Response: There does not appear to be a clear pattern of sex differences in the literature, especially when it comes to mortality due to disease. This may be tied to the difficulties in determining the true cause of death in wild tortoises. Sick tortoises may die from disease and are then scavenged by predators, or may be depredated due to lethargy from disease. We don’t often see tortoises die with direct disease causes – as they are usually confounded with drought, predators, etc. There is a signal with specific environmental conditions that females are killed by coyotes more frequently (Esque et al. 2010), but disease was not a factor in this study. We hope to address your comment in greater detail in future work on this topic.
- **[Line 406]** This is fascinating that males and females may have different immune strategies. Response: Thank you.
- **[Line 412]** In general, or in response to mycoplasma? Response: We clarified in this sentence that they are prone in general.

**Comments Received from USGS Internal Reviewer** on July 17, 2020.

Diane Elam, PhD; USGS WERC Research Manager

- **[Line 89]** Is there a word missing? After G. aggassizii? Response: We added “infection”
- **[Line 154**] Without this deletion, this is an incomplete sentence. Response: We corrected the sentence as suggested.
- **[Line 298**] This breaks up a long sentence. You need to clarify what is meant by “they all.” Is this the correct interpretation? Or is it referring to the 40? Response: We clarified this sentence. “The 5 genes differentially expressed in both comparisons were upregulated relative to control (NI) with large log2-fold changes of 14.1–16.4 (SI-NI comparison values).”
- **[Line 358]** To me, include means it’s an incomplete list. Response: We corrected this sentence to read “These sex-biased genes were identified as *TMEM135*, *ST6GALNAC4*, *IGHE*, *TRIM3*, and TRIM68-like, gopAga1_00007704, gopAga1_00017285, and gopAga1_00017523.”
- **[Line 371]** Before you were using MI and SI. Response: Since this is the first paragraph of the discussion, we reiterate the full group names and include its abbreviations as well.
- **[Line 496]** Something is wrong here. I think maybe it’s saying that “considering it warrants “further study”….odd phrasing. Response: We modified the sentence to include “Overall, the sex-biased patterns in the literature are complex and sex-biased outcomes of *Mycoplasma* spp. infection and URTD warrants further study.”
- **[Line 556]** You seem to use the acronyms sometimes. It is inconsistent. Response: We decided to keep the full group name in the conclusion section but have changed the full group name to acronyms throughout the manuscript where we felt necessary.

__________________________________________________________________________

***PLOS ONE* Journal Editor Comments**

1. Please ensure that your manuscript meets PLOS ONE's style requirements, including those for file naming. The PLOS ONE style templates can be found at [https://journals.plos.org/plosone/s/file?id=wjVg/PLOSOne_formatting_sample_main_body.pdf](https://urldefense.proofpoint.com/v2/url?u=https-3A__journals.plos.org_plosone_s_file-3Fid-3DwjVg_PLOSOne-5Fformatting-5Fsample-5Fmain-5Fbody.pdf&d=DwMFaQ&c=l45AxH-kUV29SRQusp9vYR0n1GycN4_2jInuKy6zbqQ&r=B1aK7AHqKGCeN3_0uATtsRstATg-R0cIwmIPk_jcDnE&m=2EjNJ9OBCpmMMFwY-KOYldo0JktIaay5HlbbvjyukTw&s=rATji78Ql8zDzArcStESe_hOoPmuvq2e-cPFj0JPrfQ&e=) and [https://journals.plos.org/plosone/s/file?id=ba62/PLOSOne_formatting_sample_title_authors_affiliations.pdf](https://urldefense.proofpoint.com/v2/url?u=https-3A__journals.plos.org_plosone_s_file-3Fid-3Dba62_PLOSOne-5Fformatting-5Fsample-5Ftitle-5Fauthors-5Faffiliations.pdf&d=DwMFaQ&c=l45AxH-kUV29SRQusp9vYR0n1GycN4_2jInuKy6zbqQ&r=B1aK7AHqKGCeN3_0uATtsRstATg-R0cIwmIPk_jcDnE&m=2EjNJ9OBCpmMMFwY-KOYldo0JktIaay5HlbbvjyukTw&s=LlGAOfvWitjvBL7x5BYWtVu4B4MC4Y1ZouU0aW8Iqug&e=). Response: We followed the requirements as specified.
2. To comply with PLOS ONE submissions requirements, please provide methods of sacrifice in the Methods section of your manuscript. Response: We added this information to the methods section as suggested.
3. In your Methods section, please provide additional location information of the study sites, including geographic coordinates for the data set if available. Response: We added the geographic coordinates (lat/long) as well as described the County, State, and Country in the methods.
4. We note that you are reporting an analysis of a microarray, next-generation sequencing, or deep sequencing data set. PLOS requires that authors comply with field-specific standards for preparation, recording, and deposition of data in repositories appropriate to their field. Please upload these data to a stable, public repository (such as ArrayExpress, Gene Expression Omnibus (GEO), DNA Data Bank of Japan (DDBJ), NCBI GenBank, NCBI Sequence Read Archive, or EMBL Nucleotide Sequence Database (ENA)). In your revised cover letter, please provide the relevant accession numbers that may be used to access these data. For a full list of recommended repositories, see [http://journals.plos.org/plosone/s/data-availability#loc-omics](about:blank%23loc-omics) or [http://journals.plos.org/plosone/s/data-availability#loc-sequencing](about:blank%23loc-sequencing). Response: Sequence data is available via NCBI SRA accession numbers SRS3600670 to SRS3600682 and BioSample accession numbers SAMN09727116 to SAMN09727140.
5. Thank you for stating the following in the Competing Interests section: 'I have read the journal's policy and the authors of this manuscript have the following
   competing interests: G.A.D. is a member of the Board of Directors for the Desert
   Tortoise Council, a nonprofit conservation organization that protects desert tortoises
   and their habitats. The DTC had no involvement in the design, implementation, or
   interpretation of this study.'
   - Please confirm that this does not alter your adherence to all PLOS ONE policies on sharing data and materials, by including the following statement: "This does not alter our adherence to PLOS ONE policies on sharing data and materials.” (as detailed online in our guide for authors [http://journals.plos.org/plosone/s/competing-interests](about:blank)).  If there are restrictions on sharing of data and/or materials, please state these. Please note that we cannot proceed with consideration of your article until this information has been declared. Response: We confirm it does not alter our adherence to the PLOS ONE policies on sharing data and materials.

- Please include your updated Competing Interests statement in your cover letter; we will change the online submission form on your behalf. Response: We include the Competing Interests statement in our cover letter.
- Please know it is PLOS ONE policy for corresponding authors to declare, on behalf of all authors, all potential competing interests for the purposes of transparency. PLOS defines a competing interest as anything that interferes with, or could reasonably be perceived as interfering with, the full and objective presentation, peer review, editorial decision-making, or publication of research or non-research articles submitted to one of the journals. Competing interests can be financial or non-financial, professional, or personal. Competing interests can arise in relationship to an organization or another person. Please follow this link to our website for more details on competing interests:[http://journals.plos.org/plosone/s/competing-interests](about:blank). Response: Acknowledged.

Although the decision is major, the reviewers are quite position to this research. Please address these concerns as much as possible, especially concerns raised by the reviewer 2.

1. **Comments to the Author**
   Is the manuscript technically sound, and do the data support the conclusions?
   The manuscript must describe a technically sound piece of scientific research with data that supports the conclusions. Experiments must have been conducted rigorously, with appropriate controls, replication, and sample sizes. The conclusions must be drawn appropriately based on the data presented.

- Reviewer #1: Yes
- Reviewer #2: Partly

2. Has the statistical analysis been performed appropriately and rigorously?

- Reviewer #1: Yes
- Reviewer #2: Yes

3. Have the authors made all data underlying the findings in their manuscript fully available?
   The [PLOS Data policy](https://urldefense.proofpoint.com/v2/url?u=http-3A__www.plosone.org_static_policies.action-23sharing&d=DwMGaQ&c=l45AxH-kUV29SRQusp9vYR0n1GycN4_2jInuKy6zbqQ&r=8VwICmg4nTICSP08NB49Nl0DJ6TbwaYeSfvvdlzAoAY&m=AOU1UX8WHgm07aBQDhLLJgT2UEApjn6XeSchm44uJEY&s=H0QuOhFoHXUaQFwttXfqF873M7MkQV86hahL28kSnLA&e=) requires authors to make all data underlying the findings described in their manuscript fully available without restriction, with rare exception (please refer to the Data Availability Statement in the manuscript PDF file). The data should be provided as part of the manuscript or its supporting information, or deposited to a public repository. For example, in addition to summary statistics, the data points behind means, medians and variance measures should be available. If there are restrictions on publicly sharing data—e.g. participant privacy or use of data from a third party—those must be specified.

- Reviewer #1: Yes
- Reviewer #2: Yes

4. Is the manuscript presented in an intelligible fashion and written in standard English?
   PLOS ONE does not copyedit accepted manuscripts, so the language in submitted articles must be clear, correct, and unambiguous. Any typographical or grammatical errors should be corrected at revision, so please note any specific errors here.
   Reviewer #1: Yes

Reviewer #2: Yes

5. Review Comments to the Author
   Please use the space provided to explain your answers to the questions above. You may also include additional comments for the author, including concerns about dual publication, research ethics, or publication ethics. (Please upload your review as an attachment if it exceeds 20,000 characters).

________________________________________________________________________
 **Journal Reviewer #1:** Santiago Sanchez-Ramirez.

In this study, Xu and colleagues compared blood-cell transcriptional profiles between healthy and diseased desert tortoises infected with a species-specific Mycoplasma. Besides comparing expression between treatment groups, they focused on sex-specific differences with the aim of distinguishing sex-biased immunological responses. They found that the majority of genes show sex-biased differences in expression but not to different levels of infection, for which they found a few dozen genes or less. I think the results presented here are an important contribution to different fields (i.e. genomics and evolutionary biology) and not just desert tortoise biology and conservation. The manuscript is very well written from the Methods section onwards, with the Abstract and Intro falling short, and, to my opinion, needing a bit more work. Consequentially, the two main comments that I have are:

- The Introduction is a bit short and has room for improvement. It can also benefit from having better transitions between topics. As you said, there aren't many studies about immunity in non-avian reptiles so it would be best to have a solid background on the system. There are parts in the Discussion that I feel need to be stated in the Intro as well, such as, expectations about differences between sexes and about expression in blood-cells and how it relates to immunity. Which genes are expected to be upregulated and why? Response: We expanded and revised the introduction and discussion to increase the content and manage topic transitions. We now include discussion of sex-biased gene expression and effects of sex determination in species to better set up the discussion. We added expectations about sex-biased immune responses as well.
- As I explain below, I don't find it surprising that you are finding a strong sex-biased signal. There has been a lot of work, both experimental and theoretical, explaining sex-biased differences in gene expression, and they obviously point to sexual selection and sexual conflict. Therefore, it would be great if more of this type of literature is mentioned and discussed in the manuscript. I think you do a good job at briefly mentioning this in the discussion, but there is much more to be said, and there is nothing in the intro about this. Response: This was a very good suggestion. We expanded on the literature and relevance of published work with strong sex-biased signals. We were surprised in that tortoises don’t exhibit strong physiological differences between sexes aside from sex hormones, calcium, etc. Previous studies that evaluated transcription levels for select genes also did not find a sex difference. But we agree with your point that this pattern in well documented across taxa; as such, we incorporated this in our revised manuscript.
- Other than that, I think this is a very well-done study and I'm looking forward to reading revised versions of it. I hope that you find my comments below useful.

**TITLE**

- [**Line 1**] This may be journal-dependent, but the title has some words capitalized and others don't. To make it consistent you need to capitalize "threatened", "desert", and "tortoise", unless you have a good reason not to. Response: We converted the title to sentence-case.
- The title also seems a bit broad and does not highlight your main results. You could try something along the lines: "Sex-biased expression overshadows transcriptomic immune responses to infection in the Mojave Desert tortoise". Response: We thank you for the suggestion, but after much consideration, we have decided to keep the original title.

**ABSTRACT**

- [**Line 26**] Why is this surprising? Differential expression between males and females in sexually reproducing species is absolutely widespread. There is a ton of evidence of sex-biased expression and theory supporting why this happens (i.e. sexual selection and sexual conflict). Sexually dimorphic phenotypes have been shaped by sexual selection continuously over millions of years, whereas immune responses to pathogens probably have more ephemeral and dynamic footprints on the genome. Here are some reviews on the subject [of sex-biased expression]:
  Rice WR (1984) Sex chromosomes and the evolution of sexual dimorphism. Evolution, 38, 735–742.
  Ellegren H, Parsch J (2007) The evolution of sex-biased genes and sex-biased gene expression. Nature Reviews Genetics, 8, 689–698.
  Mank JE (2017) The transcriptional architecture of phenotypic dimorphism. Nature Ecology & Evolution, 1, 1–7.
  Kasimatis KR, Nelson TC, Phillips PC (2017) Genomic Signatures of Sexual Conflict. Journal of Heredity, 108, 780–790.
  Rowe L, Chenoweth SF, Agrawal AF (2018) The genomics of sexual conflict. The American Naturalist, 192, 274–286. Response: We removed “surprising from this sentence as suggested. Thank you for the reference literature. We included them as well as other citations when appropriate and expanded on the background information for sex biased expression.
- [**Line 31**] "further investigation". Response: We edited sentence as suggested.
- [**Lines 29:32**] This statement is a bit long and convoluted. Please restructure. Perhaps into 2 sentences? It is also poorly connected to the previous sentence. Why "specifically"? Response: We revised this sentence as suggested.
- [**Line 33**]: "enriched GO [biological?] processes for". Response: This change has been made.
- [**Line 35**]: Are these all sex-biased genes or just the 8 that have sex-by-immune responses? Please clarify. Response: The 8 genes referenced here are all sex-biased genes and were also differentially expressed in the experimental immune group analysis (i.e. they are sex-biased *immune* genes).We modified this sentence for clarity.
- [**Lines 37:39**]: Instead of concluding with methodological suggestion, why don't you make a general statement about an most important take-home message of your results or why your findings are important for the scientific community? Response: We reordered parts of the abstract so it ends with suggestions for future work, which in our viewpoint seems like a natural ending place.

**INTRODUCTION**

- [**Line 42**] "particularly because of how". Response: This change has been made.
- [**Lines 43-54**]: The order of importance of these challenges is non-trivial. Availability of genomic resources seems to me to be less important than "fewer functional studies about the immunological mechanisms and pathways" for understanding innate and adaptive immune systems of non-avian reptiles. I think it makes more sense to place the genomic challenge at the end. That way you can connect the fact that more data is underway with your next paragraph on RNA-seq. Response: We kept the order as-is because it transitions naturally into the next paragraph, we don’t intent to imply they are in any particular order.
- [**Line 55**] Also check out this review paper in Nature Rev. Genetics. It quite relevant for your study:
  Klein SL, Flanagan KL (2016) Sex differences in immune responses. Nature Reviews Genetics, 1–13. Response: Thank you. We reviewed the paper and added as appropriate.
- [**Line 55**] "how sex-biased differences manifest in the immune function". This is oddly written. Sex differences may influence immune function, but it is strange to think about sex differences manifesting in the immune function. Response: This sentence was rephrased as “how the sex of the organism modulates immune functions..”
- [**Line 58**] ", such as RNA-seq, ". Response: Done.
- [**Line 59**] I don't think its the right place to introduce the system. Perhaps in a different paragraph. Why don't you move the first sentence in this paragraph to the last of the previous paragraph? Response: We added a transition sentence here to improve clarity and added detail prior to this paragraph to support the flow of this section.
- [**Lines 57-65**] This paragraph is kind of weakly constructed. Why don't you introduce the system by mentioning aspects about the biology and conservation status. Then mention that Mycoplasma research is important for conservation efforts and that there has been a lot of interest to understand host-pathogen relationships. Response: We restructured this paragraph and added additional information on the biology and conservation of tortoises and the host-pathogen relationship.
- Are there any kind of expectations about differentially expressed genes under these experimental conditions? Response: We added our expectations concerning differentially expressed genes to the last paragraph of the introduction as suggested.

**METHODS**

- [**Line 151**] "read-trimming". Response: Done.
- [**Line 159**] For rlog-transformed data did you use DSeq2? If so, this needs to be mentioned. Response: We did use DESeq2 to rlog transform the data and have added this.
- Also, log2. Write "2"in log2 as sub index. Response: Done.
- [**Line 169**]: mention false-discovery-rate before FDF. And it should be FDR < 0.05. Response: This change has been made.
- [**Line 187**] italicize "p" in p-values. Response: Done.

**Results**

- [**Line 210**] "males in the Low Infection group". Response: This sentence is referencing both males that are in the low infection group.
- [**Fig. 1**] It is important that you also highlight the puncture group, as it is a group that had significant differential expression. You have enough space to spell out the full name of the treatment groups, not just the abbreviation. It would be nice if you added vertical and horizontal axes that intercept 0. It is easier to see the variance in PCs from the center. You could also wrap around elipsoides or hull polygons to the male and female samples. It would be easier to see the sex differences that way. We thank the reviewer for the suggestion. We added axis lines and ellipsoids for sex, but we did not add the venipuncture site or ellipsoids for immune groups because there are already two variables plotted (with 2 and 3 levels, respectively), we were afraid it would make the figure too busy. We also want to focus specifically on sex and immune groups here. But, for puncture site the reader is referred to the venipuncture site PCA (S1 Fig A).
- [**Line 224**] Good. I was just about to ask that. Response: Thank you.
- I wonder how much of the gene expression signal is related to stress due to manipulation. Response: All samples were collected within 15 minutes of capture (most less than 5 mins). Previous work on this species using an ACTH challenge and subsequent corticosterone measurements indicated that additional time would be required (>30 minutes) to illicit a handling stress response (Drake et al. 2012 Animal Conservation). We don’t have any experience with this species on the time required for potential transcription changes to occur immediately after handling. Good question and something we hope to address in future work. We also note that all animals were handled similarly, so any stress response should be common across all animals.
- [**Line 249**] "log2-fold changes". Response: Done.
- [**Fig 3B**] is not referenced anywhere in the text. If it is not relevant send it to the supplementary data. Response: Figure 3B is referenced in line 534 of the track change manuscript version and line 341 of the clean manuscript version.
- [**Fig. 2**], legend: it should be alpha < 0.05, not alpha = 0.05. You will probably need to correct this throughout the manuscript. Response: Done.
- [**Line 274**] "log2-fold change". Response: Done.
- [**Line 276**] "log2-fold change". Response: Done.
- What about genes with a sex-by-immune response interaction? (i.e. sex:treatment) Response: Unfortunately, we did not analyze sex-by-immune response interactions because our sample size was not sufficient for statistical power, but agree that identifying sex-by-immune response interaction genes would be of interest.

**DISCUSSION**

- [**Line 317**]: Again, I don't think this is too surprising (see my comment on the abstract). However, it is intriguing that you have this level of differentiation between sexes in blood tissue, as this is not a sexually differentiated trait. Perhaps this is something you can discuss in the light of significant evidence and theory explaining sex-biased expression. Response: We included additional sentences to describe the differentiation between sexes in blood. In the methods we discuss that unlike mammalian blood, reptile red blood cells are nucleated and transcriptionally active (Desert et al. 2019, Waits et al., 2019). Additionally, we suggest that sex-biased differences in gene expression, particularly those with immune roles, are not detectable until after infection or immune challenge.
- [**Line 325**]: How does the results presented here directly aid future management and conservation practices? I don't see the connection. How does these kind of studies aid treatment of M. agassizii? I think it is interesting from the scientific point of view, but it is hard to see practical applications. Response: We included additional sentences in this section to describe previous work that attempted to use genetic responses to quality and quantify animal responses that are responding to specific environmental (changes in diet, drought) or biological (disease) processes. Our finding in this paper are leading managers to a better understanding of how tortoises immunologically respond to this pathogen and disease, and hopefully result in improve diagnostics and surveillance protocols for the species. In addition, the 5 DEGs that scale with infection may be suitable biomarkers to test for age/stage of infection. Finally, our male-specific results—1) that males may be seasonally immunocompromised, and 2) that they may be pathogen reservoirs because they interact with more individual and maintain larger ranges (and this may translate to greater gene expression variance) suggests that timing of studies and of translocations may be important.
- [**Lines 329-331**]: This or a version of this should be in the intro. Response: We reworded the introduction to include this information.
- [**Line 338**] "studies on turtles"? Response: Change made.
- [**Line 341**]: "In our data,". Response: Done.
- [**Line 353**]: This should also be in the intro. Expectations about experiments, and why. Response: We added a similar sentence to the introduction to describe our experimental expectations.
- [**Line 361**]: What do you mean with "previously described"? In another paper or in the results section? Clarify. Response: We clarified this in the sentence.
- [**Line 263**]: "log2-fold change". Response: Done.
- [**Line 364**]: "immunity-related differentially expressed genes". Response: We revised this phrase to differentially expressed immune genes.
- [**Lines 379-381**]: This is ambiguous. Are you referring to your own results or the results of other studies? Response: Yes, we are referring to our results and clarified this in the sentence.
- [**Lines 385**]: Citation? Response: We added this citation.
- [**Line 388**]: "increasingly starting to be recognized"? Response: Done.
- [**Lines 392-393**]: This should be in the intro as part of your expectations.
  I wonder to what extent hormones play a role in triggering different sex-biased transcriptomic profiles. For example, differences in (mammal) prostaglandin levels can probably trigger sex-biased responses to inflammation and healing. Response: We updated the introduction and included a new paragraph on the potential role of hormones in our work.
- [**Line 433**]: "In this study, ". Response: Done.
- [**Line 434**]: "GO" instead of "Gene Ontology". Just to be consistent. Response: Done.
- [**Line 435**]: "tortoises, as expected.". Response: Change made.
- [**Line 437**]: What about sex-specific seasonal variation? You can imagine that as you approach reproductive season hormone levels in males might be geared towards finding mate and perhaps male-male competition, whereas in in females the would be geared towards nesting and resource allocations. Just an idea. Response: We reorganized this section to address the role of female nesting behavior, etc.

______________________________________________________________________________

**Journal Reviewer #2:**

- This is a novel and interesting use of RNA sequencing to understand differences in diseased and healthy tortoises. Therefore, I commend acceptance, but I suggest some additional references to put this study into a more accurate ecological context, and one important caveat of interpretations in the Discussion. Response: We thank the reviewer for their time to comment and improve this work.
- The main caveat of interpreting this study is that we now know that most tortoises with M. agassizii infections, have very low loads, with no signs of disease, and no adaptive antibody responses. In particular, recent work (Braun et al. 2014, Sandmeier et al. 2017) showed that many animals positive by qPCR for M. agassizii, but do not have a positive antibody response. Similarly, Weitzman et al. (2017) and Sandmeier et al. (2018) showed that many tortoises tested weakly positive for M. agassizii, without showing any signs of URTD. Aiello et al. (2016, 2018) also confirmed that the load of M. agassizii appears to be associated with signs of URTD and adaptive antibody responses, and that adaptive immune responses can take weeks-months to be induced. Therefore, animals with induced antibodies have been experiencing infections over a significant time-frame. Response: Thank you for pointing this out. We added a paragraph in the introduction to summarize these points.
- Therefore, I do not think this study was set up to detect differences in innate immunity, which would control such low-load infections. Innate immunity needs to be overcome, in a sense, to stimulate and adaptive antibody response. Therefore, even the “low” infection group has been diseased for a long time and already stimulated the adaptive immune system in the form of induced antibodies. In natural populations, prevalence of induced antibodies in Mojave tortoises is actually relatively low (Sandmeier et al. 2013). Response: We reviewed and referenced Sandmeier et al. 2013 and others and included this information in the introduction. We agree that even “low” infected animals had been infected for ~4 years prior to our sampling. Even though this low infected group had acquired antibodies by the time of our sampling, so it is likely they were using portions of both the innate and adaptive system for protection. We capture this we modified text throughout to refer to both innate and adaptive immune responses.
- Obviously, it would be hard/impossible to add a whole other group of tortoises to this study, but it should be addressed that even the “low infection” group in fact presents with much higher morbidity than the average naturally-occurring tortoises with low loads of M. agassizii and no signs of morbidity/adaptive immune responses. Calling groups Medium and Severe Infection, and stating in the introduction that no carrier/subclinical/low infection animals were used would be more accurate. Because many of these studies came out after your samples were collected, I see no fault with the conceptualized study design at the time, but only in retrospect. Response: We renamed the low infection group “medium infection” as suggested. In retrospect, we would redesign this experiment to capture responses frequently after exposure/infection to capture those truly low infected animals. We added the following sentence in the introduction to improve clarity as suggested . “Higher pathogen loads from *M. agassizii* generally correlate with increased clinical signs and adaptive antibody responses (Aiello et al., 2016; Aiello et al., 2018); however, antibody production can be delayed in this species by months to years after *M. agassizi* (Aiello et al., 2018; Drake et al., 2019). By the time most tortoises are classified has having URTD and an antibody response is detected, individuals have likely been infected for a long period of time. For some time initially after infection by pathogen, tortoises may not show clinical signs or an antibody response but would test positive for *M. agassizii* by qPCR (Braun et al., 2014; Sandmeier et al., 2017; Weitzman et al., 2017). As many of the recovery unit populations remain below viability levels [32], it is important to the health and viability of the species to understand how these bacterial infections and URTD impact the health of tortoises.” We also include the following statement in the methods. “In our study, both medium and severe infection groups were sampled from captive colonies with documented infection for multiple years. We were not able to incorporate subclinical animals with early or low levels of infection in this study.”
- In particular, I suggest the following changes:
  **COMMENTS:**
- [**Line 60**] M. agassizii is not known to be intracellular – and the reference (Jacobson et al. 2014) does not claim that this is well understood. Response: We removed “intracellular” from this sentence. The Jacobson et al. paper described tortoise mycoplasmosis are one of the most extensively characterized infectious diseases of chelonians. We modified our sentence to include “Infection of the Mojave desert tortoise (*Gopherus agassizii*) by the pathogenic bacteria *Mycoplasma* spp. and subsequent disease are among the most extensively characterized in chelonians.
- [**Line 66**]: We know M. agassizii is associated with the mucosal epithelium: we do not know how/if the microbe binds to cells. Many respiratory microbes also are just found within mucosal layer, and not bound to epithelial cells. Response: We removed “bind to” from this sentence and added the following sentence for clarity. “Although this disease has been extensively studied in desert tortoises, it remains unclear if or how *M. agassizii* bind to epithelial cells or if they just reside in the mucosal layer similar to other respiratory microbes (Saab et al. 2014)”.
- [**Line 73**]: Add “adaptive and” innate immune response (why just the innate response?) Again, I think the study design is not ideal to detecting changes in innate responses during very early/low load infections. Response: Thank you we have changed this throughout.
- [**Line 82**]: Here and thoughout: change “Mycoplasmal” to lower case – “mycoplasma” is a generic term that refers to bacteria within the Mollicutes. It should only be capitalized when it is used as a genus name. Throughout: do not use Mycoplasma to refer to M. agassizii. Response: Changes made throughout the manuscript.
- **[Line 334**]: Reference 1 is listed twice. Response: Duplicate reference removed.
- [**Line 345-347**]: This study followed turtles after an experimental infection. Therefore, the investigators likely caught that early, innate response which your study likely missed. Response: You are correct in that study on Chinese soft-shelled turtles used a bacterial challenge experiment and measured immediate innate responses.
- [**Line 364**]: Reword to “differentially expressed immune genes”. Response: Done.
- [**Line 416-418**]: I have not seen substantial evidence of sex-biased susceptibility to M. agassizii. In fact, other studies have failed to detect differences (Sandmeier et al. 2013). An alternate interpretation is that sex-biased differences change seasonally – which is also the case for levels of immune cells and functions in tortoises and other reptiles (e.g. Zimmerman et al. 2010, Sandmeier et al. 2016, Goessling et al. 2017). Response: We thank the reviewer for this observation and have included this hypothesis in our discussion. While Sandmeier et al., 2016 did not find sex differences in immune cell levels, this study was conducted in desert tortoises with no previous history of infection or URTD. Therefore, we also proposed that sex differences in immune cells and immune response may present only after infection and immune challenge.
- [**Line 426-428**]: These statements seem irrelevant. In fact, the mechanism described cannot occur in animals without sex chromosomes! Make a clearer statement and suggest that sex hormones are likely the driving mechanism of sex differences. Response: We agree with this comment, our point was that sex-biased gene expression is studied primarily on sex chromosomes, which are absent in tortoises. This is therefore additional motivation for why sex-biased gene expression in a tortoise is interesting. We moved this information to our introduction and clarified this intention. We have also clarified our statement in the discussion suggesting that sex hormones are critical modulators of sex-biased gene expression and immunity.
- [**Line 439-440**]: This statement needs a reference. Much of this behavior starts in April/May in Mojave desert tortoise populations. Response: We modified this sentence to include April – mid July and referenced Turner et al. 1986.
- [**Line 455-456**]: However, females also invest a large amount of energy in producing eggs – female energy expenditure in producing offspring is often downplayed in the literature, but should not be! At this time of year, most females would have already laid eggs. Possibly, they are just depleted of iron? Response: Good point! We added text to describe this. “Additionally female tortoises lay their eggs in April-mid July (Turner et al. 1986), which requires a large investment of energy and resources. Allocation of energetic resources during this period may deplete iron and could further contribute to transcriptional differences related to iron homeostasis between males and females.”
- [**Line 480**]: It is incorrect to say that late summer is the “mating season” of desert tortoises. Response: We revised this sentence to include the following, “Because tortoises have temperature based sex determination, effects of sex hormones are of primary interest, including the potential immunosuppressive effect of testosterone and corticosterone during select periods of their activity”.
- Additional references (need not all be cited, but provide additional information on the epidemiology of URTD in natural/seminatural populations):
  a. Aiello CM, TC Esque, KE Nussear, PG Emblidge, JP Hudson. 2018. The slow dynamics of mycoplasma infections in a tortoise host reveal heterogeneity pertinent to pathogen transmission and monitoring. Epidemiology and Infection 147:e12.
  b. Braun, J., M. Schrenzel, C. Witte, L. Gokool, J. Burchell, and B. Rideout. 2014. Molecular methods to detect Mycoplasma spp. and testudinid herpesvirus 2 in desert tortoises (Gopherus agassizii) and implication for disease management. Journal of Wildlife Diseases 50:757-766.
  c. Goessling, JM, SA Koler, BD Overman, E Hiltbold, C Guyer, MT Mendoca. 2017. Lag of Immunity across seasonal acclimation states in gopher tortoises (Gopherus polyphemus). J of Exper Zool 327:235-42.
  d. Sandmeier FC, Horn KR, Tracy CR. 2016. Temperature-independent, seasonal fluctuations in immune function of the Mojave desert tortoise (Gopherus agassizii). Canadian Journal of Zoology 94:583-590.
  e. Sandmeier, F.C., C.R. Tracy, B.E. Hagerty, S. DuPré, H. Mohammadpour, and K. Hunter, Jr. 2013. Mycoplasmal upper respiratory tract disease across the range of the threatened Mojave desert tortoise: associations with thermal regime and natural antibodies. E
     Sandmeier FC, Weitzman CL, Tracy CR. 2018. An ecoimmunological approach to disease in tortoises reveals the importance of lymphocytes. Ecosphere. 9:e02427.
  f. Sandmeier FC, Weitzman CL, Maloney KN, Tracy CR, Nieto N, Teglas MB, Hunter KW, DuPré S, Gienger CM, Tuma MW. 2017. Comparison of current methods for the detection of chronic mycoplasmal URD in wild populations of the Mojave desert tortoise (Gopherus agassizii). Journal of Wildlife Diseases 53:91-101.
  g. Weitzman, C.L., F.C. Sandmeier, and C.R. Tracy. 2017b. Prevalence of the upper respiratory pathogen Mycoplasma agassizii in Mojave desert tortoises (Gopherus agassizii). Herpetologica 73:113-120.

Response: Thank you for the suggestions, we found these references useful. We reviewed and incorporated these references and others throughout the manuscript as appropriate.
